# Harnessing microbial volatiles to replace pesticides and fertilizers

**DOI:** 10.1111/1751-7915.13645

**Published:** 2020-08-07

**Authors:** Gareth Thomas, David Withall, Michael Birkett

**Affiliations:** ^1^ Biointeractions and Crop Protection Department Rothamsted Research Harpenden Hertfordshire AL5 2JQ UK; ^2^ School of Biosciences College of Life and Environmental Sciences University of Exeter Exeter EX4 4QD UK; ^3^ Present address: Microbiomes, Microbes and Informatics Group, Organisms and Environment Division School of Biosciences Cardiff University Cardiff Wales CF10 3AX UK

## Abstract

Global agricultural systems are under increasing pressure to deliver sufficient, healthy food for a growing population. Seasonal inputs, including synthetic pesticides and fertilizers, are applied to crops to reduce losses by pathogens, and enhance crop biomass, although their production and application can also incur several economic and environmental penalties. New solutions are therefore urgently required to enhance crop yield whilst reducing dependence on these seasonal inputs. Volatile Organic Compounds (VOCs) produced by soil microorganisms may provide alternative, sustainable solutions, due to their ability to inhibit plant pathogens, induce plant resistance against pathogens and enhance plant growth promotion. This review will highlight recent advances in our understanding of the biological activities of microbial VOCs (mVOCs), providing perspectives on research required to develop them into viable alternatives to current unsustainable seasonal inputs. This can identify potential new avenues for mVOC research and stimulate discussion across the academic community and agri‐business sector.

## Graphical abstract

Pesticides and fertilizers are applied to agricultural crops to reduce losses by pathogens, and enhance crop biomass, although their production and application can also incur several economic and environmental penalties. Microbial Volatile Organic Compounds (mVOCs) could provide more sustainable alternatives to these inputs, due to their ability to inhibit plant pathogens, induce plant resistance against pathogens, and enhance plant growth promotion. This review will highlight recent advances in our understanding of the biological activities of mVOCs, highlighting their potential suitability as a more sustainable alternative to pesticides and fertilizers.

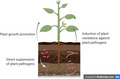

## Introduction

By 2100, the United Nations projects that the global population will increase by around 4 billion, which may require agricultural production to double or triple to keep pace with population growth (United Nations Department of Economic and Social Affairs Population Division, 2017; Rohr *et al*., 2019). To date, agricultural practice has relied on the application of synthetic chemical inputs to optimize crop yields, including synthetic pesticides, which reduce crop losses by targeting plant pathogens, and synthetic fertilizers, applied to increase crop biomass. Synthetic pesticides play a critical role in mitigating crop damage by pathogens, which are responsible for annual crop losses of 17–30% for the five major crops (Savary *et al*., 2019). However, the development of synthetic pesticides is in itself unsustainable, estimated to cost approximately $250 million to bring a single active ingredient to market, with an estimated success rate of 1 in 140 000 synthesized compounds (Lamberth *et al*., 2013). Moreover, the over‐application of pesticides can lead to the development of pesticide resistance, rendering them less effective. The production and application of inorganic nitrogen fertilizer has resulted in crop production being the largest cause of human alteration to the global nitrogen cycle (Smil, 1999). The Haber–Bosch process is used to produce inorganic nitrogen fertilizer, through the conversion of hydrogen and nitrogen into ammonia. However, this process is energy intensive, occurring at high temperatures and pressure and generating a carbon footprint contributing ~1.2% of overall global anthropogenic CO_2_ emissions (Nørskov and Chen, 2016). Furthermore, the application of inorganic nitrogen to soils leads to enhanced microbial production of nitrous oxide (N_2_O), the potent greenhouse gas, through soil microbial nitrification and de‐nitrification. As such, concentrations of N_2_O have substantially increased in the atmosphere since 1960 as a direct result of fertilizer applications (Davidson, 2009). With projected increases in crop demand, agricultural expansion could result in approximately 10‐fold increases in pesticide use, and 2.7‐fold increases in fertilizer application (Rohr *et al*., 2019). Concerted efforts should therefore be made to develop more sustainable control methods to reduce over‐reliance on synthetic fertilizer and pesticides, through shifts in agronomic practice (Tester and Langridge, 2010; Fisher *et al*., 2018). Whilst genetically modified crops demonstrating enhanced disease resistance show potential to reduce pathogen damage and could potentially reduce the requirement for pesticide inputs, the regulatory frameworks required to commercialize the crops are lengthy (Kanchiswamy *et al*., 2015a). Therefore, it is an opportune time to explore alternative control strategies to chemical inputs or genetic modification.

**Fig. 1:**
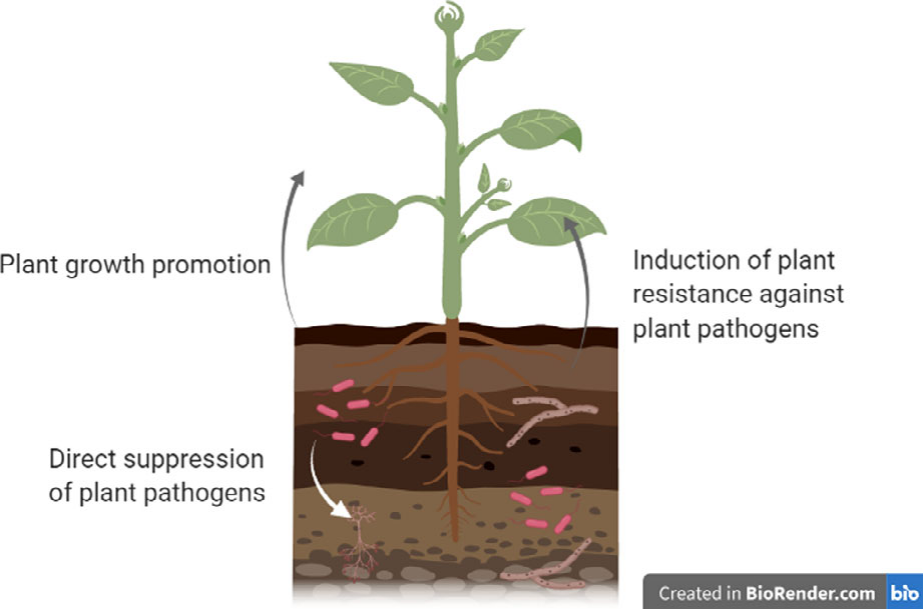
Overview of the biological activities of microbial Volatile Organic Compounds (mVOCs).

One alternative solution to chemical inputs is through the addition of antagonistic, beneficial, microorganisms, due to their ability to antagonize pathogenic soil microbes, and enhances plant biomass. Soil microorganisms produce a wide spectrum of secondary metabolites enabling them to compete with neighbouring microorganisms, which they have likely evolved to compete for the same resources within soil (Brakhage and Schroeckh, 2011; Garbeva and Weisskopf, 2020). For example, bacteria from the genus of soil‐dwelling *Streptomyces* spp. produce a diverse range of secondary metabolites, which have been exploited for human medicine, with approximately 80% of antibiotics currently being sourced from the genus (de Lima Procópio *et al*., 2012). The structural diversity of secondary metabolites explains their broad spectrum of activities, including mediating communication intra‐ and inter‐specifically, defence against competitors, nutrient acquisition and symbiotic interactions (Spiteller, 2015; Macheleidt *et al*., 2016). Whilst most research on microbial secondary metabolites focusses on non‐volatile compounds, increasing attention is being paid to microbial volatile organic compounds (mVOCs). VOCs are a class of secondary metabolites with a low molecular weight (< 300 Da), high vapour pressure and low boiling points, which tend to be lipophilic in nature (Schulz‐Bohm *et al*., 2017). Their ability to diffuse through gas and water‐filled pores within the heterogenous soil matrix make them suitable for both short‐ and long‐distance signalling (Maffei *et al*., 2011; Kanchiswamy *et al*., 2015b; Schulz‐Bohm *et al*., 2017). Under competitive soil conditions, due to the presence of other competing organisms, VOCs are important for antibiosis and signalling for symbiotic interactions (Effmert *et al*., 2012). The capability of mVOCs to suppress neighbouring pathogens and signal to plants demonstrates their potential to be exploited as alternatives to chemical fertilizers and pesticides, which could provide a more sustainable solution, as well as having negligible hazardous effects on animals and the environment (Tilocca *et al*., 2020). This review focuses on the role of mVOCs in maintaining plant health, through the direct suppression of plant pathogens, the induction of plant resistance against pathogens and the promotion of plant growth (Fig. 1), highlighting their potential as alternative solutions to synthetic pesticides and fertilizers.

## Role of volatiles in the suppression of plant pathogens

Several studies demonstrate mVOCs can inhibit a range of plant pathogens, highlighting their suitability as a potential sustainable alternative to pesticides. One of the first examples demonstrating an inhibitory role for mVOCs against plant pathogens were those produced by *Pseudomonas* species isolated from soybean and canola, in the inhibition of *Sclerotinia sclerotiorum*; a fungal pathogen with a broad host range of over 400 plant species (Fernando *et al*., 2005). Of 23 VOCs identified from *Pseudomonas* species, six significantly reduced mycelial growth of *S. sclerotiorum*. Similarly, VOC production by two strains of *Bacillus* endophytes significantly reduced the weight and number of the vegetative, long‐term survival structures (sclerotia) of *S. sclerotiorum* (Massawe *et al*., 2018). VOCs from *Burkholderia ambifaria* (Groenhagen *et al*., 2013) and a range of other rhizobacterial isolates (Velivelli *et al*., 2015) have also demonstrated the ability to inhibit growth of the ubiquitous soil‐borne pathogen *Rhizoctonia solani*. MVOCs can also display inhibitory activity against bacterial pathogens. Exposure of *Clavibacter michiganensis*, the causal agent of bacterial ring rot of potato, to VOCs from *Bacillus subtilis* led to significant inhibition of pathogen growth, with benzaldehyde, nonanal, benzothiazole and acetophenone specifically demonstrating inhibitory activities (Rajer *et al*., 2017). *Bacillus* VOCs also inhibited the growth of *Xanthomonas oryzae*, the causal agent of bacterial leaf blight of rice, with decyl alcohol and 3,5,5‐trimethylhexanol specifically inhibiting pathogen growth (Xie *et al*., 2018). As well as inhibition against fungal and bacterial pathogens, mVOCs can display inhibitory activity against pathogenic oomycetes. Exposure of *Phytophthora capsici* to *Bacillus* and *Acinetobacter* VOCs significantly reduced mycelial growth of the oomycete, with 3‐methyl‐1‐butanol, isovaleraldehyde, isovaleric acid, 2‐ethylhexanol and 2‐heptanone showing specific inhibitory roles (Syed‐Ab‐Rahman *et al*., 2019). VOCs produced by *Nodulisporium* also demonstrated anti‐oomycete activity against several *Pythium* species, although the causal VOCs involved in this inhibition were not individually assayed (Sánchez‐Fernández *et al*., 2016). These studies highlight mVOCs can display inhibitory activity against a range of fungal, bacterial and oomycete pathogens, which could have biotechnological potential as alternatives to pesticides. A summary of the individual VOCs involved in pathogen suppression from the studies discussed is presented in Table 1.

**Table 1:** Summary of mVOC producing stains, their active VOCs and their target pathogens.

VOC producing strain	Active VOCs	Target pathogen	Reference
Bacteria			
*Bacillus amyloliquefaciens* FZB42,	Benzaldehyde	*Ralstonia solanacearum*	Tahir and colleagues (2017a)
*Bacillus artrophaeus* LSSC22	1,2‐Benzisothiazol‐3(2H)‐one		
	1,3‐Butadiene		
*Bacillus subtilis* FA26	Benzaldehyde	*Clavibacter michiganensis*	Rajer and colleagues (2017)
	Nonanal		
	Benzothiazole		
	Acetophenone		
*Bacillus* spp. (VM10, VM11, VM42)	2‐Undecanone	*Sclerotinia sclerotiorum*	Massawe and colleagues (2018)
	1,3‐Butadiene		
	Benzothiazole		
	N,N‐Dimethyldodecylamine		
*Bacillus* strain D13	Decyl alcohol	*Xanthomonas oryzae*	Xie and colleagues (2018)
	3,5,5‐Trimethylhexanol		
*Bacillus amyloliquefaciens* UQ154	3‐Methyl‐1‐butanol	*Phytophthora capsici*	Syed‐Ab‐Rahman and colleagues (2019)
*Bacillus velezensis* UQ156	Isovaleric acid		
*Acinetobacter* spp. UQ202	2‐Ethylhexanol		
	2‐Heptanone		
	Isovaleraldehyde		
*Burkholderia ambifaria*	2‐Undecanone	*Rhizoctonia solani*	Groenhagen and colleagues (2013)
	4‐Octanone	*Alternaria alternata*	
	Dimethyl trisulfide		
	S‐Methyl methanethiosulfonate		
	2‐Propylacetophenone		
	Dimethyl disulfide		
*Penicillium expansum*	(*R*)‐(−)‐1‐Octen‐3‐ol	*Penicillium chrysogenum*	Yin *et al*., 2019
*Penicillium solitum*			
*Penicillium paneum*			
*Pseudomonas fluorescens*	Benzothiazole	*Sclerotinia sclerotiorum*	Fernando and colleagues (2005)
*Pseudomonas chloroaphis*	Cyclohexanol		
*Pseudomonas aurantiaca*	n‐Decanal		
	Dimethyl trisulfide		
	2‐Ethyl 1‐hexanol		
	Nonanal		
Rhizobacterial isolates	2,4‐Di‐tert‐butylphenol	*Rhizoctonia solani*	Velivelli and colleagues (2015)
	2‐Hexen‐1‐ol		
*Streptomyces* spp.	Caryolan‐1‐ol	*Botrytis cinerea*	Cho and colleagues (2017)
*Streptomyces alboflavus*	Not identified	*Aspergillus flavus*	Yang and colleagues (2019)
Fungi			
*Nodulisporium* sp. GS4dII1a	Not identified	*Pythium aphanidermatum*	Sánchez‐Fernández and colleagues (2016)
*Saccharomyces cerevisiae*	3‐Methyl‐1‐butanol	*Colletotrichum gloeosporoides*	Rezende and colleagues (2015)
	2‐Methyl‐1‐butanol	*Colletotrichum acutatum*	
*Trichoderma* spp.	Not identified	*Sclerotinia sclerotiorum*	Ojaghian and colleagues (2019)

Due to the presence of a chiral centre, 1‐octen‐3‐ol has two optical isomers: (*R*)‐(−)‐1‐octen‐3‐ol and (*S*)‐(+)‐1‐octen‐3‐ol. Interestingly, when these optical isomers were investigated for inhibitory roles against the fruit spoilage pathogen *Penicillium chrysogenum*, (*R*)‐(−)‐1‐octen‐3‐ol inhibited spore germination of five out of seven isolates, whereas (*S*)‐(+)‐1‐octen‐3‐ol inhibited spore germination of only two isolates, suggesting the different enantiomers display differences in inhibitory activities (Yin *et al*., 2019). Furthermore, (*R*)‐(−)‐1‐octen‐3‐ol modulated the transcription of a greater number of genes in *Penicillium chrysogenum*. This highlights an important consideration in the specificity of mVOCs for target pathogens, providing a potential avenue for future research in the investigation of the bioactivity of chiral VOCs, as well as providing chemical structural information for the development of active substances to replace pesticides.

Whilst the role of mVOCs in the suppression of plant pathogens is well established, the molecular mechanisms involved in their inhibitory activities are receiving increasing attention. When exposed to *Bacillus* VOCs, the tomato wilt pathogen *Ralstonia solanacearum* showed a reduction in the expression of a range of virulence factor genes, including those related to chemotaxis, type 3 and type 4 secretion systems, and extracellular polysaccharides, as well as increasing the expression of a global virulence factor (Tahir *et al*., 2017a). Specifically, benzaldehyde, 1,2‐benzisothiazol‐3(2H)‐one and 1,3‐butadiene produced by *Bacillus* were involved in the modulation of virulence factor expression of the pathogen. Similarly, expression of genes involved in virulence and biofilm formation in *Xanthomonas oryzae* were also downregulated upon exposure to *Bacillus* VOCs (Xie *et al*., 2018). VOCs produced by *Streptomyces* spp. inhibited the production of aflatoxins from the fungal pathogen *Aspergillus flavus*, through the downregulation of several genes involved in aflatoxin biosynthesis (Yang *et al*., 2019; Lyu *et al*., Lyu^2020^). Exposure of *Sclerotinia sclerotiorum* to VOCs produced by *Trichoderma* species led to the upregulation of four glutathione S‐transferase genes, which are involved in the detoxification of antifungal secondary metabolites, which may contribute to the virulence of *Sclerotinia sclerotiorum* (Ojaghian *et al*., 2019). Sphingolipid metabolic processes, vesicle formation and trafficking, and membrane localization were all disrupted upon exposure of *Botrytis cinerea* to the *Streptomyces*‐derived VOC caryolan‐1‐ol (Cho *et al*., 2017). Plasma membrane disruption of pathogens has also been observed upon exposure of *Colleotrichum* species to the yeast derived VOCs 3‐methyl‐1‐butanol and 2‐methyl‐1‐butanol, leading to increased electrolyte loss (Rezende *et al*., 2015). Whilst the modes of action underpinning pathogen suppression by mVOCs are receiving increasing attention, a greater understanding of their molecular targets across a broader range of pathogenic microorganisms is critical prior to their deployment into open fields.

## Role of volatiles in induced resistance

As well as directly suppressing plant pathogens, mVOCs can induce plant resistance to pathogens, where plant defences are preconditioned by prior treatment, resulting in enhanced resistance and reducing susceptibility to plant diseases. This was first observed by Ryu and colleagues (2004), who exposed *Arabidopsis thaliana* seedlings to *Bacillius* VOCs, which reduced the severity of symptoms by the soft‐rot causing bacterial pathogen *Erwinia carotovora*. Seedlings exposed to VOCs produced by strains deficient in 2,3‐butanediol and acetoin biosynthesis developed greater disease symptoms relative to wild‐type strain VOCs, suggesting a specific role for these VOCs in induced systemic resistance. These findings have been extended under greenhouse conditions, where exposure of cucumber to 2,3‐butanediol led to enhanced resistance against the bacterial pathogen *Pseudomonas syringae* (Song *et al*., 2019b). Interestingly, specificity in the ability of the different isomers of 2,3‐butanediol to induce plant resistance have also been observed, with (2*R*, 3*R*)‐butanediol inducing resistance of tobacco against *Erwinia carotovorus*, whereas (2*S*, 3*S*)‐butanediol was ineffective (Han *et al*., 2006). Whilst most work on mVOCs in induced resistance has focussed on 2,3‐butanediol and acetoin, 3‐pentanol and 2‐butanone have also been shown to induce resistance of cucumber against *Pseudomonas syringae*, and albuterol and 1,3‐butadiene play a role in the induction of resistance of tobacco against *Ralstonia solanacearum* (Song and Ryu, 2013; Tahir *et al*., 2017b). As stomata can act as entry points for bacterial invasion, mVOCs may induce stomatal closure to reduce pathogen internalization. This was investigated by Wu and colleagues (2018), who demonstrated that exposure of *A. thaliana* and tobacco to 2,3‐butanediol and acetoin induced stomatal closure, although the influence of stomatal closure on pathogen populations was not determined.

Fungal VOCs have also demonstrated a role in inducing plant resistance against pathogens. *A. thaliana* seedlings exposed to *Trichoderma virens* VOCs demonstrated significantly reduced disease symptoms when inoculated with *Botrytis cinerea*, and symptoms were greater in seedlings exposed to a *Trichoderma virens* mutant deficient in sesquiterpene production, suggesting a role for sesquiterpenes in induced resistance (Contreras‐Cornejo *et al*., 2014). Exposure of *A. thaliana* seedlings to 6‐pentyl‐2H‐pyran‐2‐one, a VOC commonly produced by a range of *Trichoderma* species (Jeleń *et al*., 2014), demonstrated significant reductions in disease symptoms when inoculated with the fungal pathogens *Botrytis cinerea* and *Alternaria brassicicola* (Kottb *et al*., 2015). 1‐Octen‐3‐ol, another commonly reported fungal‐derived VOC, elicited *A. thaliana* defence responses against *Botrytis cinerea* (Kishimoto *et al*., 2007), although as this was tested as a racemic mixture, the role of the two optical isomers of 1‐octen‐3‐ol in induced resistance cannot be discerned. More recently, VOC production from archaea (*Nitrosocosmicus oleophilus*), which have received little attention relative to bacteria and fungi, have also been shown to induce systemic resistance of *A. thaliana* against *Pseudomonas syringae* and *Pectobacterium carotovorum*; a necrotrophic bacterium responsible for soft‐rot of a range of vegetables (Song *et al*., 2019a). This suggests the biotechnological potential for mVOCs in sustainable agriculture is not limited to bacteria and fungi, and archaea may provide a new avenue for future research. A summary of the individual VOCs involved in induced resistance from the studies discussed is presented in Table 2.

**Table 2:** Summary of mVOC producing stains, their active VOCs, the plants displaying induced resistance upon VOC exposure and the target pathogens.

VOC producing strain	Active VOCs	Target pathogen/plant species	Reference
Bacteria			
*Ampleomyces* sp. F‐a‐3	Methyl benzoate	*Pseudomonas syringae/A. thaliana*	Naznin and colleagues (2014)
*Bacillus subtilis* GB03	2,3‐Butanediol	*Erwinia carotovora/A. thaliana*	Ryu and colleagues (2004)
*Bacillus amyloliquefaciens* IN937a	Acetoin		
*Bacillus* spp.	3‐Pentanol	*Pseudomonas syringae/Cucumis sativus*	Song and Ryu (2013)
	2‐Butanone		Song and colleagues (2015)
*Bacillus amyloliquefaciens* FZB42	Benzaldehyde	*Ralstonia solanacearum/Nicotiana benthamiana*	Tahir and colleagues (2017a)
*Bacillus artrophaeus* LSSC22	1,2‐Benzisothiazol‐3(2H)‐one		
	1,3‐Butadiene		
*Bacillus subtilis* SYST2	Albuterol	*Ralstonia solanacearum*/*Nicotiana benthamiana*	Tahir and colleagues (2017b)
	1,3‐Propanediol		
*Bacillus amyloliquefaciens* FZB42	2,3‐Butanediol	*A. thaliana/Nicotiana benthamiana*	Wu and colleagues (2018)
	Acetoin		
*Bacillus subtilis* GB03	2,3‐Butanediol	*Pseudomonas syringae/Cucumis sativa*	Song and colleagues (2019b)
	Acetoin		
*Paenibacillus polymyxa* E681	Tridecane	*Pseudomonas syringae/A. thaliana*	Lee and colleagues (2012)
*Pseudomonas chlororaphis* O6	(2*R*, 3*R*)‐Butanediol	*Erwinia carotovora/Nicotiana benthamiana*	Han and colleagues (2006)
Fungi			
*Cladosporium* sp. D‐c‐4	M‐Cresol	*Pseudomonas syringae/A. thaliana*	Naznin and colleagues (2014)
*Talaromyces wortmannii* FS2	β‐Caryophyllene	*Colletotrichum higginsianum/Brassica campestris*	Yamagiwa and colleagues (2011)
*Trichoderma virens*	Terpenes	*Botrytis cinerea/A. thaliana*	Contreras‐Cornejo and colleagues (2014)
*Trichoderma asperellum*	6‐Pentyl‐2H‐pyran‐2‐one	*Botrytis cinerea, Alternaria brassicicola/A. thaliana*	Kottb and colleagues (2015)
Archaea			
*Nitrosocosmicus oleophilus* MY3	Not identified	*Pectobacterium carotovorum, Pseudomonas syringae/A. thaliana*	Song and colleagues (2019a)
Exogenous application			
N.A.	1‐Octen‐3‐ol	*Botrytis cinerea/A. thaliana*	Kishimoto and colleagues (2007)
N.A.	Dimethyl disulfide	*Sclerotinia minor/*Tomato	Tyagi and colleagues (2020)

Several studies indicate mVOCs induce resistance of plants against pathogens through the regulation of plant hormones, which can be specifically elicited by different mVOCs. Plant defences are modulated by two main resistance pathways. Induced systemic resistance is mediated by jasmonic acid and ethylene, and commonly associated with beneficial microbes (Pieterse *et al*., 2014), and systemic acquired resistance, mediated by salicylic acid and commonly induced by pathogens (Shine *et al*., 2019). However, in some cases, beneficial microbes can trigger salicylic acid dependent induced systemic resistance (Pieterse *et al*., 2014). Induced systemic resistance of *A. thaliana* against *Erwinia carotovorans* by *Bacillus subtilis* GB03 VOCs was dependent on ethylene biosynthesis, although induced resistance by *Bacillus amyloliquefaciens* IN937A was independent of ethylene signalling, suggesting different VOCs present in the blends may utilize alternative pathways to induce resistance (Ryu *et al*., 2004). Contrastingly, resistance of cucumber to *Pseudomonas syringae* exposed to *Bacillus subtilis* GB03 VOCs involved jasmonic acid, but not ethylene signalling (Song *et al*., 2019b). Discrepancies in these findings may relate to differences in plant species under investigation, which may utilize different defence pathways in VOC perception, or redundancy in salicylic acid, jasmonic acid and ethylene signalling pathways in induced resistance (Ryu *et al*., 2004). A role for jasmonic acid signalling has also been observed in 3‐pentanol and 2‐butanone induced resistance, although expression of salicylic acid and ethylene marker genes were not induced (Song and Ryu, 2013). Similarly, *A. thaliana* mutants exposed to 3‐pentanol confirmed 3‐pentanol mediated immune response involved jasmonic acid and salicylic acid signalling pathways, as well as the non‐pathogenesis related 1 (*NPR‐1*) gene, but that ethylene signalling genes were not involved (Song *et al*., 2015). Tridecane, produced by *Paenibacillus polymyxa* E681, was involved in the enhanced resistance of *A. thaliana* after pathogen challenge with *Pseudomonas syringae*, through the modulation of salicylic acid and jasmonic acid marker genes (Lee *et al*., 2012). The *Bacillus* VOCs albuterol and 1,3‐propanediol enhanced defences of tobacco against *Ralstonia solanacearum* by inducing expression of resistance (*RRS1*) and pathogenesis‐related proteins (*Pr1a* and *Pr1b*), which act as markers for salicylic acid signalling (Tahir *et al*., 2017b). Interestingly, 1,3‐propanediol induced greater expression of the *RRS1* gene relative to albuterol, whereas albuterol induced greater expression of pathogenesis‐related (*PR*) genes, suggesting specificity in the mechanisms of induced resistance for the VOCs. Similar specificity in VOC induction was observed by Naznin and colleagues (2014), who demonstrated M‐cresol, the dominant VOC from *Cladosporium*, induced salicylic acid and jasmonic acid signalling pathways in *A. thaliana* when challenge inoculated with *Pseudomonas syringae*, whereas methyl benzoate, the dominant VOC from *Ampleomyces*, induced jasmonic acid signalling with partial salicylic acid signals. Expression of genes involved in salicylic acid signalling is also induced in tomato plants exposed to dimethyl disulfide, enhancing defence against *Sclerotinia minor* (Tyagi *et al*., 2020). Interestingly, as well as directly suppressing growth of *Ralstonia solanacearum*, benzaldehyde, 1,2‐benzisothiazol‐3(2H)‐one and 1,3‐butadiene elicited induced systemic resistance in tobacco, through induction in the transcriptional expression of defence related genes, demonstrating potential multi‐functional roles of mVOCs (Tahir *et al*., 2017a).

## Role of volatiles in plant growth promotion

Microbial VOCs also have the potential to enhance plant growth, enabling them to potentially be exploited as a new category of fertilizer, previously described as ‘gaseous fertilizer’ (Sharifi and Ryu, 2018). The role of mVOCs in promoting plant growth has been recognized for over a decade and was first reported by Ryu and colleagues (2003). *A. thaliana* seedlings exposed to VOCs of *Bacillus subtilis* GB03 and *Bacillus amyloliquefaciens* IN937a showed enhancements in leaf area, for which 2,3‐butanediol and acetoin demonstrated a role when applied exogenously. Since this, VOCs from several species of *Bacillus* have shown a role in plant growth promotion. VOCs from a different strain of *Bacillus subtilis* (SYS2) also promoted growth of tomato, for which albuterol and 1,3‐propanediol played a specific role (Tahir *et al*., 2017c), suggesting different strains of the same species of *Bacillus* can deploy different VOCs to enhance plant growth. 2‐Pentylfuran, produced by cultures of *Bacillus megaterium*, demonstrated dose‐dependent growth promotion of *A. thaliana*, with an approximate 1.5‐fold increase in plant biomass observed at a 10 µg dose (Zou *et al*., 2010). As well as *Bacillus* spp., VOCs produced by other rhizobacteria can enhance plant growth, including *Proteus vulgaris*, which enhanced plant growth of Chinese cabbage, for which indole demonstrated a role (Yu and Lee, 2013). Groenhagen and colleagues (2013) also observed significant increases in *A. thaliana* biomass when exposed to a range of VOCs, with dimethyl disulfide, the most abundantly produced VOC across a range of *Burkholderia ambifaria* strains, demonstrating the greatest plant growth promoting effects between doses of 1 ng and 1 mg.

Several fungal VOCs have also demonstrated a role in plant growth promotion, with 6‐pentyl‐2H‐pyran‐2‐one from *Trichoderma* spp. shown specifically to influence plant growth. *A. thaliana* seedlings exposed to 6‐pentyl‐2H‐pyran‐2‐one demonstrated a reduction in fresh plant weight, but also a reduction in disease symptoms when inoculated with certain fungal pathogens (Kottb *et al*., 2015). Contrastingly, Garnica‐Vergara and colleagues (2016) showed the application of 6‐pentyl‐2H‐pyran‐2‐one led to increased biomass and root branching of *A. thaliana* between 50 and 175 µM, although at the highest tested doses, a phytotoxic effect was observed. Discrepancies in the findings between these studies are likely due to differences in the doses and methods of application of 6‐pentyl‐2H‐pyran‐2‐one used in each study. Whilst 6‐pentyl‐2H‐pyran‐2‐one is the most well‐studied *Trichoderma* VOC, evidence suggests other VOCs may also be involved in plant growth promotion. Exposure of *A. thaliana* to VOCs from a range of *Trichoderma* species showed 6‐pentyl‐2H‐pyran‐2‐one production was reported from certain strains which did not promote plant growth and was not produced by certain strains which did, suggesting other VOCs could contribute to the growth promotion observed. (Lee *et al*., 2016). This is supported by findings from Estrada‐Rivera and colleagues (2019), who showed that 2‐heptanol, 3‐octanol and 2‐heptanone produced by *Trichoderma atroviride* can also promote plant growth of *A. thaliana*. VOCs from other fungal species have also demonstrated roles in plant growth promotion, including *Fusarium oxysporum*, which significantly enhanced lettuce biomass, with β‐caryophyllene demonstrating a specific role in growth promotion (Minerdi *et al*., 2011). Interestingly, β‐caryophyllene enhanced the biomass of *Brassica campestris*, as well as inducing resistance against *Colletotrichum higginsianum* (Yamagiwa *et al*., 2011). A summary of the individual VOCs involved in plant growth promotion from the studies discussed is presented in Table 3.

**Table 3:** Summary of mVOC producing stains, their active VOCs and the plants displaying enhanced growth promotion upon VOC exposure.

VOC producing strain	Active VOCs	Plant species	Reference
Bacteria			
*Bacillus subtilis* GB03	2,3‐Butanediol	*A. thaliana*	Ryu and colleagues (2003)
*Bacillus amyloliquefaciens* IN937a	Acetoin		
*Bacillus megaterium* XTBG34	2‐Pentylfuran	*A. thaliana*	Zou and colleagues (2010)
*Bacillus subtilis* SYST2	Albuterol	Tomato (*Solanum lycopersicum*)	Tahir and colleagues (2017c)
	1,3‐Propanediol		
*Burkholderia ambifaria*	Dimethyl disulfide	*A. thaliana*	Groenhagen and colleagues (2013)
	Acetophenone		
	3‐Hexanone		
Fungi			
*Fusarium oxysporum*	β‐Caryophyllene	Lettuce (*Lactuca sativa*)	Minerdi and colleagues (2011)
*Proteus vulgaris*	Indole	Chinese cabbage (*Brassica rapa*)	Yu and Lee (2013)
*Trichoderma virens*	6‐Pentyl‐2H‐pyran‐2‐one	*A. thaliana*	Garnica‐Vergara and colleagues (2016)
*Trichoderma* spp.	1‐Decene	*A. thaliana*	Lee and colleagues (2019)
*Trichoderma atroviride*	6‐Pentyl‐2H‐pyran‐2‐one	*A. thaliana*	Estrada‐Rivera and colleagues (2019)
	2‐Heptanol		
	3‐Octanol		
	2‐Heptanone		
Exogenous application			
N.A.	Dimethyl disulfide	*A. thaliana*	Tyagi and colleagues (2019)

Several studies indicate mVOCs may promote plant growth through modulating plant hormone responses. The *cytokinin‐ and ethylene‐insensitive 2* (*ein‐2*) and *Arabidopsis cytokinin receptor‐deficient 1* (*cre‐1*) mutants exposed to *Bacillus subtilis* GB03 VOCs did not display increases in plant biomass, suggesting a role for cytokinin signalling pathways plant growth promotion (Ryu *et al*., 2003). *ein‐2* also demonstrated a role in the growth promotion of *A. thaliana* by the VOC 6‐pentyl‐2H‐pyran‐2‐one, as well as auxin transport proteins (Garnica‐Vergara *et al*., 2016). Exposure of *A. thaliana* to 1‐decene, a plant growth promoting *Trichoderma* VOC, led to the differential expression of 123 genes, 17 of which were upregulated and several of which were auxin related (Lee *et al*., 2019). Similarly, dimethyl disulfide altered the root system architecture of *A. thaliana*, which were dependent on canonical auxin signalling pathways, with mutants deficient in auxin responsive genes and transcription factors not exhibiting lateral root development or growth enhancement (Tyagi *et al*., 2019).

## Field applications of VOCs

For mVOCs to serve as an alternative to synthetic pesticides and fertilizers, it is important to determine the efficacy of active VOCs under open‐field conditions. Dimethyl disulfide is a VOC produced by bacteria including *Bacillus cereus*, which can suppress soil‐borne pathogens and nematodes, and elicit systemic resistance against *Botrytis cinerea* and *Cochliobolus hetereostrophus* (Huang *et al*., 2012). Dimethyl disulfide has been successfully commercialized as an alternative to pesticides as the soil fumigant PALADIN^®^, which has been patented (Paladin Technical EPA Reg. No. 55050‐3), highlighting the potential of mVOCs to serve as alternatives to chemical inputs (de Boer *et al*., 2019). Performance of other VOCs demonstrating a role in induced plant resistance under laboratory conditions, which commonly occur in Petri dish environments, are also demonstrating promise in the field and under soil conditions. Field trials with 2,3‐butanediol induced resistance of cucumber to viruses (Kong *et al*., 2018) and maize to the northern corn leaf blight fungus *Setosphaeria turcica* under a soil context (D’Alessandro *et al*., 2014). As well as 2,3‐butanediol, cucumber plants exposed to 3‐pentanol and 2‐butanone showed reduced disease symptoms against the *Pseudomonas syringae* under open‐field conditions (Song and Ryu, 2013). These studies demonstrate promise in the performance of mVOCs in the field, and future work should investigate the efficacy of bioactive VOCs identified from laboratory‐based studies under field conditions.

## Conclusions and future outlook

The biological activities of mVOCs highlight their potential to act as alternatives to unsustainable agricultural chemical inputs, to feed a growing population. So far, much work investigating mVOCs focusses on the model plant species *A. thaliana* and *Nicotiana benthamiana* (Tables 2 and 3), and therefore, future research should focus on the protective and growth stimulating effects of mVOCs on crop and vegetable species. Similarly, characterization of mVOCs has been performed on limited range of microbial species. In terms of bacteria, *Bacillus* spp., in particular 2,3‐butanediol and acetoin, have been the focus of several studies, and for fungi, *Trichoderma* species has attracted the most attention, specifically 6‐pentyl‐2H‐pyran‐2‐one (Tables 2 and 3). Current estimates indicate that < 10% of mVOCs have been ascribed a function (Lemfack *et al*., 2018), suggesting enormous potential for identifying other mVOCs with biotechnological applications. Moreover, most studies reported here investigate VOC production from axenic cultures of microbes, although growing bodies of evidence suggest interspecific interactions between microorganisms can enhance production of VOCs which have demonstrated inhibitory activity against pathogens (Tyc et al., 2014, 2017). This could enable identification of biologically relevant VOCs involved in the suppression of pathogenic microorganisms. Whilst several studies also investigate the role of mVOCs on a single biological activity, there are likely overlaps in the roles of these VOCs. For example, 6‐pentyl‐2H‐pyran‐2‐one has demonstrated roles in pathogen suppression (e.g. Jeleń *et al*., 2014), plant growth promotion (Garnica‐Vergara *et al*., 2016), and induced resistance (Kottb *et al*., 2015), suggesting biological activities should not be considered in isolation. Moreover, whilst many studies demonstrate VOCs have suppressive effects on plant pathogens, it is important to determine the effect of these inhibitory VOCs on plant development. For example, inhibitory mVOCs produced by *Streptomyces yanglinensis* 3–10 against *Aspergillus* were tested to determine their effects on plant development and showed that VOCs did not inhibit peanut seedling germination, suggesting promise for use under field conditions (Lyu *et al*., 2020). The modes of action of VOCs in the suppression of target pathogens (Dalilla *et al*., 2015; Cho *et al*., 2017; Tahir *et al*., 2017a; Xie *et al*., 2018; Yang *et al*., 2019; Ojaghian *et al*., 2019), enhanced disease resistance of plants (Ryu *et al*., 2004; Lee *et al*., 2012; Song and Ryu, 2013; Tahir *et al*., 2017a; Tahir *et al*., 2017b; Song *et al*., 2019b; Tyagi *et al*., 2020) and plant growth promotion (Ryu *et al*., 2003; Garnica‐Vergara *et al*., 2016; Lee *et al*., 2019; Tyagi *et al*., 2019) are receiving increasing attention, future research priority should focus on understanding the mode of action of biologically active VOCs on target plants and pathogens. Whilst investigation of the efficacy of VOCs under field conditions has demonstrated promise, a wider range of VOCs require testing at this scale. More research on methods of application of mVOCs onto fields is also required, for example, the effectiveness of drench versus spraying application (Garbeva and Weisskopf, 2020). The potential for plant production of active VOCs for the biological control of fungal pathogens through companion cropping systems is another potential form of delivery. Bean cultivars resistant to *Colletotrichum lindemuthianum*, the causal agent of black spot disease, enhanced resistance of susceptible cultivars to the pathogen when exposed to VOCs from resistant cultivars (Quintana‐Rodriguez *et al*., 2015). These findings could be translated in the field for the control of plant pathogens, through companion cropping systems, using VOCs from disease‐resistant cultivars to deliver VOCs to neighbouring crops to enhance disease resistance against fungal pathogens. In conclusion, studies reviewed here demonstrate mVOCs can be exploited to serve as sustainable alternatives to agricultural chemical inputs, which can potentially reduce our overreliance on the current unsustainable methods at a time when population growth, and food demand, is likely to substantially increase.

## Funding information

Rothamsted International (Grant/Award Number: BBS/OS/CP/000001); Biotechnology and Biological Sciences Research Council (Grant/Award Number: 1622285).

## Conflict of interest

None declared.
